# Draft Genome Sequence of *Botryosphaeria dothidea*, the Pathogen of Apple Ring Rot

**DOI:** 10.1128/genomeA.01142-16

**Published:** 2016-10-27

**Authors:** Zhaotao Liu, Sen Lian, Baohua Li, Hongyun Lu, Xiangli Dong, Caixia Wang

**Affiliations:** a College of Crop Protection and Agronomy, Qingdao Agricultural University, and Key Lab of Integrated Crop Pests Management of Shandong Province, Qingdao, Shandong, People’s Republic of China; b Library of Qingdao Agricultural University, Qingdao, Shandong, People’s Republic of China

## Abstract

*Botryosphaeria dothidea* is a destructive pathogen infecting apple (*Malus domestica*) on the fruit and stem. Here, we present the draft genome sequence of *B. dothidea* (strain LW030101) using Illumina sequencing. The draft genome sequence provides useful information and acts as a platform for further research on the pathogen.

## GENOME ANNOUNCEMENT

*Botryosphaeria dothidea*, causing apple ring rot, is a destructive pathogen on apple (*Malus domestica*) worldwide, such as in China, Japan, South Korea, the United States, Australia, and South Africa. Not only does it cause damage on branches or lead to stem canker, apple ring rot also damages apple fruit, resulting in reduced quality and seriously affecting economic benefit. Guo et al. investigated 88 apple orchards, and 77.6% of the apple trees were infected by *B. dothidea* in China (1).

*B. dothidea* strain LW030101 was isolated from an experimental station at Qingdao Agricultural University, Laiyang, Shandong Province, China. Genomic DNA was isolated using the sodium dodecyl sulfate (SDS) method (2). The total genomic DNA obtained was subjected to quality control by agarose gel electrophoresis and quantified by Qubit. The genome of LW030101 was sequenced using an Illumina HiSeq 2500 by a PE125 strategy, and the sequence reads were 5.88 Gbp in raw data, with an average coverage of 103.6×. Illumina PCR adapter reads and low-quality reads were filtered, and 5.38 Gbp clean data were extracted from the raw data, for which the Q20 was 96.89% and Q30 was 92.01%. The filtered reads were assembled by SOAP*denovo* (3) (http://soap.genomics.org.cn/soapdenovo.html) to generate scaffolds. The draft genome of *B. dothidea* consists of 1,216 sequence scaffolds with a total length of 45.26 Mbp (*N*_50_, 287,735 bp; *N*_90_, 26,758 bp), 53.08% G+C content, and a maximum scaffold size of 1,260,241 bp.

The completeness of the assembly was assessed using CEGMA version 2.4 (4), which estimated the genome sequence to be 98.39% complete. Gene prediction was performed on the *B. dothidea* genome assembly by GeneMarkS (5) (http://topaz.gatech.edu/) with an integrated model combining the GeneMarkS generated (native) and Heuristic model parameters. Overall, 10,411 protein-coding gene models were predicted in the genome, with the gene total lengths of 13,151,400 bp and average gene length of 1,263 bp.

A whole-genome Blast search (*E* value <1e^−5^, minimal alignment length percentage larger than 40%) was performed against six databases. The predicted proteins that had sequence similarity to proteins in Kyoto Encyclopedia of Genes and Genomes (KEGG) (6), Clusters of Orthologous Groups (COG) (7), nonredundant (NR) protein databases, Swiss-Prot (8), Gene Ontology (GO) (9), and TrEMBL (8) were 3,662, 4,756, 9,375, 3,133, 6,775, and 9,502 proteins, respectively.

In this study, we presented the draft genome sequence of *B. dothidea*, an economic important pathogen to apple production. The sequence represents a new resource that will be useful for further research into the biology, ecology, and evolution of these key pathogens.

### Accession number(s).

This whole-genome shotgun project has been deposited at DDBJ/ENA/GenBank under the accession no. MDSR00000000. The version described in this paper is version MDSR01000000.

## References

[1] Guo L, Li J, Li B, Zhang X, Zhou Z, Li G, Wang Y, Li X, Huang L, Sun G, Wen Y 2009 Investigations on the occurrence and chemical control of *Botryosphaeria* canker of apple in China. Plant Protect 35:120–123.

[2] Lõoke M, Kristjuhan K, Kristjuhan A 2011 Extraction of genomic DNA from yeasts for PCR-based applications. Biotechniques 50:325–328. doi:10.2144/000113672. 21548894 10.2144/000113672 PMC3182553

[3] Li R, Zhu H, Ruan J, Qian W, Fang X, Shi Z, Li Y, Li S, Shan G, Kristiansen K, Li S, Yang H, Wang J, Wang J 2010 *De novo* assembly of human genomes with massively parallel short read sequencing. Genome Res 20:265–272. doi:10.1101/gr.097261.109. 20019144 PMC2813482

[4] Parra G, Bradnam K, Korf I 2007 CEGMA: a pipeline to accurately annotate core genes in eukaryotic genomes. Bioinformatics 23:1061–1067. doi:10.1093/bioinformatics/btm071. 17332020

[5] Besemer J, Lomsadze A, Borodovsky M 2001 GeneMarkS: a self-training method for prediction of gene starts in microbial genomes. Implications for finding sequence motifs in regulatory regions. Nucleic Acids Res 29:2607–2618. doi:10.1093/nar/29.12.2607. 11410670 PMC55746

[6] Kanehisa M, Goto S, Kawashima S, Okuno Y, Hattori M 2004 The KEGG resource for deciphering the genome. Nucleic Acids Res 32:D277–D280. doi:10.1093/nar/gkh063. 14681412 PMC308797

[7] Tatusov RL, Fedorova ND, Jackson JD, Jacobs AR, Kiryutin B, Koonin EV, Krylov DM, Mazumder R, Mekhedov SL, Nikolskaya AN, Rao BS, Smirnov S, Sverdlov AV, Vasudevan S, Wolf YI, Yin JJ, Natale DA 2003 The COG database: an updated version includes eukaryotes. BMC Bioinformatics 4:41. doi:10.1186/1471-2105-4-41. 12969510 PMC222959

[8] Magrane M, Consortium U. 2011 UniProt Knowledgebase: a hub of integrated protein data. Database (Oxford) 2011:bar009. doi:10.1093/database/bar009. 21447597 PMC3070428

[9] Ashburner M, Ball CA, Blake JA, Botstein D, Butler H, Cherry JM, Davis AP, Dolinski K, Dwight SS, Eppig JT, Harris MA, Hill DP, Issel-Tarver L, Kasarskis A, Lewis S, Matese JC, Richardson JE, Ringwald M, Rubin GM, Sherlock G 2000 Gene ontology: tool for the unification of biology. Nat Genet 25:25–29. doi:10.1038/75556. 10802651 PMC3037419

